# Ontogenetic thermal and metabolic patterns guide physiologically informed, size-based fishery management

**DOI:** 10.1093/conphys/coag026

**Published:** 2026-04-22

**Authors:** Lauren Stewart, Emily Ball, Clive Trueman, Jamie Stevens

**Affiliations:** Biosciences, College of Life and Environmental Sciences, University of Exeter, Hatherly Laboratories, Prince of Wales Road, Exeter EX4 4PS, UK; Ocean and Earth Science, University of Southampton, Southampton SO143ZH, UK; Ocean and Earth Science, University of Southampton, Southampton SO143ZH, UK; Biosciences, College of Life and Environmental Sciences, University of Exeter, Hatherly Laboratories, Prince of Wales Road, Exeter EX4 4PS, UK

**Keywords:** Metabolic rate, ontogeny, otolith, salmon aquaculture, stable isotopes, wrasse

## Abstract

Wrasse species are widely used as cleaner fish in salmon aquaculture but their performance as cleaner fish may be impacted by thermal and ontogenetic changes. Understanding the conditions in which each species of wrasse is most productive as cleaner fish is hugely valuable to make informed decisions on the management of fisheries. To do this, we use a novel geochemical proxy to reconstruct ontogenetic trends in thermal habitat and field metabolic rate for six species of wrasse from two sampling locations from the Isle of Skye (Scotland) and Dorset (southern England). We describe consistent ontogenetic transitions from warmer to cooler thermal habitats across all species, and demonstrate reductions in activity levels associated with ontogenetic habitat shifts after accounting for body size and temperature effects. Field metabolic rates were not significantly different between Dorset and Skye after accounting for body size and temperature. Ontogenetic shifts in thermal habitat and activity level have implications for the effectiveness of wrasse as cleanerfish, as well as providing evidence to support size-based harvesting rules. More broadly, this work highlights the potential of otolith isotope analyses to recover field-based, context-specific data on physiology and thermal ecology, advancing fisheries conservation and management strategies.

## Introduction

Thermal preference and metabolic performance are key determinants of fitness in wild fish, as ectothermic physiology links body temperature to environmental conditions. Thermal preference shapes habitat use, distribution and behaviour by allowing individuals to occupy temperatures that optimize physiological efficiency and reduce stress. While metabolic performance determines energy allocation for growth, locomotion and reproduction, setting thermal limits beyond which performance declines (Pörtner and Farrell, 2008; Pörtner  *et al*., 2017). Together, these traits influence species distributions, population resilience and ecological interactions, making thermal physiology critical for fisheries management and conservation (Cooke *et al.,* 2014; Dahlke *et al.,* 2020).

Thermal physiology also varies across ontogeny, with different life stages exhibiting distinct thermal preferences, metabolic capacities and tolerance limits (Pörtner and Peck, 2010). Juveniles, subadults and adults therefore respond differently to environmental temperatures, making some stages more vulnerable to global change and anthropogenic pressures such as habitat degradation, pollution and overfishing (Dahlke *et al.,* 2020). Despite its importance, substantial gaps remain in understanding stage-specific thermal physiology, particularly for commercially important species, which may lead to suboptimal management and reduced recruitment, growth, and survival (Pörtner and Peck, 2010; Cooke *et al.,* 2014). Addressing these gaps is essential for effective conservation, sustainable harvesting and adaptive management under changing environmental conditions.

In recent years fisheries for temperate wrasse species, have developed in multiple locations around Britain, Ireland and Scandinavia (Skiftesvik *et al*., 2014*a*; Riley *et al.,* 2017; Henly *et al.,* 2021); these species include ballan (*Labrus bergylta),* corkwing (*Symphodus melops*), cuckoo (*Labrus mixtus*), goldsinny (*Ctenolabrus rupestris*) and rock cook (*Centrolabrus exoletus*). These wrasse are used as part of parasitic louse control strategies in salmonid aquaculture (Bjordal, 1991; Gonzalez and de Boer, 2017). The ongoing removal of wrasse is anticipated to result in a range of impacts: from localized stock depletion and changes in demographic structure of populations (Halvorsen, 2017; Halvorsen *et al.,* 2017; Henly *et al.,* 2021), to indirect impacts on other taxonomic groups and the overall functioning of inshore-reef ecosystems (Figueiredo *et al.,* 2005; Norderhaug *et al.,* 2005; Henly, 2023). These species share a common habitat of inshore temperate reefs along the northeast Atlantic coast. They are associated with rock and algal cover, and are all carnivorous grazing on epibiota. Despite these similarities, they exhibit a variety of life history traits and behavioural characteristics (summarized in Henly, 2023). These differences are presumably linked to variation in physiology among species (Henly *et al*., 2024). Wrasse produce benthic eggs guarded by parents, except for the Goldsinny species, which produces planktonic eggs. Ballan and Cuckoo wrasse attain larger sizes and are protogynous hermaphrodites. A summary of life history characteristics of European wrasse is provided by Darwall *et al*. (1992). The thermal tolerance of wrasse species overlaps optimal growing temperatures for Atlantic salmon during summer (e.g. intrinsic thermal tolerance ranges). For Ballon wrasse, this was determined as 7.9–16.8°C (Palma *et al*., 2025). Optimal temperatures for salmon aquaculture are ~8–14°C, and in Scottish lochs where the majority of UK salmon aquaculture occurs, temperatures reach 14–15°C in summer. However, in winter, temperatures in salmon aquaculture cages in Scotland fall <6°C, limiting cleaning activities for wrasse (Palma *et al*., 2025). The limited movement of wrasse and limited larval drift also implies potential for population-level genetic adaptation to local thermal and ecological conditions. Local ecophysiological adaptation could have implications for performance in salmonid aquaculture as the thermal ranges of salmon and wrasse species show limited overlap. Cold-adapted wrasse populations may therefore tolerate conditions suitable for salmon aquaculture more effectively.

Metabolic rate (MR) is the rate at which an organism uses energy to maintain basic physiological functions, grow, move and reproduce (Treberg *et al*., 2016; Henly, 2023). Field metabolic rate (FMR) is the total energy expenditure of an organism in its natural environment, encompassing all daily activities and environmental challenges (Trueman *et al*., 2023). Recently it has been demonstrated that individual-level, time-averaged FMR and experienced temperature can be estimated retrospectively and simultaneously from the stable isotope composition of otoliths (Jamieson *et al*., 2004; Trueman *et al*., 2014; Sinnatamby *et al*., 2015; Chung *et al*., 2019*a*; Smoliński *et al*., 2021; Groot *et al*., 2024). Otoliths grow incrementally by precipitating layers of the calcium carbonate mineral aragonite, with newest growth on the outermost edge. Carbon (as bicarbonate) in the blood is a mixture of carbon released from cellular respiration of food and carbon derived from dissolved inorganic carbonate (DIC) from seawater exchanged across the gills. Systematic and large isotopic differences between internal respired and external dissolved carbon sources mean that the proportion of respiratory carbon in blood (and thus the otolith) can be estimated from isotopic mass balance, if the isotopic composition of otolith and sources are known or can be estimated (Kalish, 1991; Solomon *et al*., 2006; Trueman *et al*., 2016). Systematic and predictable relationships between carbon isotope compositions of otoliths and alternative proxies for metabolic rates have been demonstrated (Kalish, 1991; Sherwood and Rose, 2003; Trueman *et al*., 2014, 2016; Chung *et al*., 2019*a*; Martino *et al*., 2020; Alewijnse, 2023; Henly, 2023).

Otolith aragonite forms in near isotopic equilibrium with surrounding water, allowing experienced temperatures to be reconstructed from oxygen isotope ratios (Campana and Thorrold, 2001; Morissette *et al*., 2023). Oxygen and carbon isotope ratios can be determined simultaneously via isotope ratio mass spectrometry, requiring as little as 10 μg of aragonite powder (Jones *et al*., 2023; Groot *et al*., 2024). With knowledge of the isotopic composition of the surrounding water, time-integrated records of temperature and FMR can be obtained through otolith isotope analysis. Here, we exploit otolith-based thermometry and respirometry to reconstruct ontogenetic trends in thermal preference and field metabolic rates in coastal wrasse species across two regions with emerging fisheries conservation requirements.

In this study, we use otolith isotope-derived estimates of experienced temperature and FMR for individual wrasse to (i) describe and compare species-level ontogenetic shifts and interspecific differences in experienced temperature and energetic costs within a common site, (ii) quantify the relationship between experienced temperature and expressed FMR to assess the thermal adaptability and metabolic performance of temperate wrasses from two discrete and thermally distinct locations and (iii) assess whether location influences FMR of individuals with larger body sizes.

## Materials and Methods

### Sample collection and preparation

**Figure 1:**
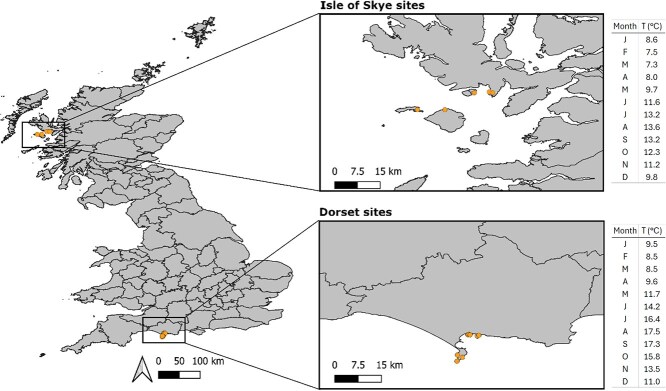
Map showing the locations of sampling in the Isle of Skye and Dorset and the average monthly sea surface temperatures over the past 10 years (watertemperature.info, 2024). Map projection: WGS84.

Sample collection was conducted in mid-September and mid-October 2019 in two locations. The first on fishing grounds between Portland and Ringsted in Dorset, southern England (hereafter ‘Dorset’) and the second on fishing grounds between Elgol and Soay on the Isle of Skye, Scotland (hereafter ‘Skye’) see Fig. 1. In the fisheries industry it is common for wrasse to be caught in Dorset for use in salmon farms in Skye, which is why these two localities were chosen. We expect there to be a roughly 2°C difference in temperature between the two localities (watertemperature.info, 2024).

Samples of the five wrasse species, namely *L. bergylta*, *S. melops*, *L. mixtus*, *C. rupestris* and *C. exoletus*, were collected from Dorset and the Isle of Skye (see Supplementary Table S1). A sixth species commonly found in southern England (Baillon’s wrasse *S. bailloni*), the use of which is unknown in salmonoid aquaculture, was also collected from Dorset to understand the full breadth of interspecific differences at this site. The total lengths (cm) of fish were measured for each individual and converted to body mass estimates using the species-specific length–weight relationships for wrasse reported in Silva *et al*. (2013). Samples were collected in association with commercial fishing operations in locations frequently targeted by wrasse fishermen. Wrasse were caught in depths <10 m using either rod-and-line or in pots over the course of 1 day (Isle of Skye) or 3 days (Dorset), due to operational differences between fishers in each location. Dorset fish were sampled before size-based sorting for individuals for fishers to retain while Skye fish were sampled after sorting. Permission from the relevant management authorities was obtained to collect samples above and below the voluntary minimum and maximum conservation reference sizes (CRS). For each individual, the total length (cm) was recorded and the fish was killed using an approved Schedule 1 method in compliance with UK Home Office Scientific Procedures (Animals) Act Requirements and the University of Exeter’s Ethics Policy.

Whole fish were placed onto ice as soon as possible and transferred to a −20°C freezer at the earliest opportunity. Within species, fish were pseudo-randomly selected to give adequate and approximately equal representation of sizes across the size range of captured fish. Sagittal otoliths and a sample of dorsal white muscle tissue (~1 cm^3^) were extracted from the selected fish. Skin and remaining bones were removed from muscle tissue before being stored at −20°C until freeze-drying. Otoliths were washed in distilled water to remove remaining tissue and left to air dry. Cleaned otoliths were fixed onto a backing plate sulcus-side upwards and milled across the sulcal ridge surfaces of the proximal side. This was achieved using a dremel engraving tool with a medium diamond bit to obtain aragonite powder from the most recently formed aragonite on the external surface of the otolith. Muscle tissue samples were freeze-dried at −57°C overnight, and crushed to a fine homogenous powder using a pestle and mortar.

### Mass spectrometry

Approximately 25 μg of powder from each otolith sample was weighed accurately into borosilicate reaction vessels and analysed at the Stable Isotope Ratio Mass Spectrometry Laboratory, Southampton, UK, using a Kiel IV carbonate device coupled to a MAT253 isotope ratio mass spectrometer (both Thermo Fisher Scientific, Bremen, Germany). The samples were reacted with 106% phosphoric acid for 600 s at 70°C. Water vapour and other gas traces were removed cryogenically and the remaining CO_2_ was measured several times against a reference gas. Following data reduction and corrections, data were normalized using a two-point calibration with NBS 18 (International Atomic Energy Agency, Vienna, Austria) and GS1 (in-lab reference material previously calibrated against NBS 18 and NBS 19). Isotope ratios for C and O in otolith aragonite are reported in delta (δ) notation relative to Vienna Pee Dee Belemnite. In-house reference material (Solnhofen limestone, Germany) was used for quality assurance purposes and to report instrument precision. Long-term instrument precision is 0.03‰ for δ^13^C and 0.06‰ for δ^18^O.

Subsamples of 0.8–1.2 mg of muscle tissue were weighed into 6 × 4 mm tin capsules and successively analysed for δ^13^C using a Thermo Fisher Scientific Isolink elemental analyser interfaced with a Delta V Plus isotope ratio mass spectrometer. This was carried out at the Natural Environmental Isotope Facility (NEIF) Stable Isotope Ecology Laboratory (SEIL) at the Scottish Universities Environmental Research Centre (SUERC) between October 2022 and January 2023. International and internal reference materials were placed at the start of each run (~90–100 samples). International reference materials were USGS88, USGS43, USGS91 and USGS42 (RSIL, 2019, 2020a, 2020b). Internal reference materials were placed every 8–10 samples to correct for instrumental drift, and to characterize and correct any linearity effects (Werner and Brand, 2001).

### Data analysis

All analyses were carried out in R statistical software version 4.2. or later (R Core Team, 2024).

### Calculation of experienced temperature using δ ^18^O

Otolith δ^18^O values were used to reconstruct the time-averaged temperature experienced by each individual using the following equation (Urey *et al*., 1951):


(1) \begin{equation*} \mathrm{Temperature}\ \left({}^{{}^{\circ}}C\right)=\frac{\left({\delta}^{18}{O}_{oto}-{\delta}^{18}{O}_{sw}\right)-a}{-b} \end{equation*}

δ^18^O*_oto_* is the oxygen isotope ratio in the sampled otolith and δ^18^O*_sw_* is the oxygen isotope ratio in seawater. δ^18^O*_sw_* were estimated from the compiled dataset LOCEAN (Reverdin *et al*., 2022), compiling measured δ^18^O obtained within 1.5 degrees of latitude and longitude from the fish sampling locations and restricted to salinities in excess of 30 a.s.u. δ^18^O*_sw_* values for Dorset and Skye were set at 0.5‰ and 0.3‰, respectively. The terms *a* and *b* in equation (1) are coefficients from the linear relationship between oxygen isotope fractionation between water and otolith aragonite (δ^18^O_oto_–δ^18^O_SW_) and temperature, which was set according to the generalized δ^18^O temperature-dependent fractionation equation for saltwater fish (Morissette *et al*., 2023), so that the intercept, *a* = 3.465 and the slope, *b* = −0.209.

### Exploring ontogenetic variations in experienced temperature

For the wrasse from Dorset, for which a broad range of body sizes were available (total length (TL) 6–48 cm), we explored ontogenetic variation in experienced temperature and how this varied across species. Using a linear model, log_10_(body mass), species and the interaction between these two variables were included as predictors of experienced temperature. Tukey tests with *P*-values adjusted for multiple comparisons were used to test for significant differences between species in the final model. We also contrasted the experienced thermal niches for body sizes above and below the CRS size limits.

### **Calculation of** FMR **using δ**^**13**^**C**

The proportion of respiratory carbon in the fish’s blood derived from otolith carbonate (C_resp_) was estimated using the following mass balance equation from (Chung *et al*., 2019*b*):


(2) \begin{equation*} {C}_{resp}=\frac{\left({\delta}^{13}{C}_{oto}-{\delta}^{13}{C}_{DIC}\right)}{\left({\delta}^{13}{C}_{diet}-{\delta}^{13}{C}_{DIC}\right)}+\varepsilon \end{equation*}

where δ^13^C_oto_ is the carbon isotope ratio in the sampled otolith. δ^13^C_diet_ was determined from muscle δ^13^C from the same individuals. Where muscle tissue δ^13^C were not available for an individual, the average δ^13^C from the remaining individuals of that species was used (Henly *et al*., 2024). δ^13^C values from muscle tissue were not lipid extracted or corrected, as δ^13^C_diet_ aims to capture the δ^13^C of all respired carbon, including that from lipids. δ^13^C_DIC_ is the average δ^13^C of DIC in seawater. As the δ^13^C of seawater was not measured at the time of sampling, this value was estimated as the observed δ ^13^C_DIC_ for the North Sea (1‰) as identified in Burt *et al*. (2016). The ε term, the total net isotopic fractionation from the carbon sources to the otolith carbonate, was set at zero (Solomon *et al*., 2006; Chung *et al*., 2019*a*).

C_resp_ values represent a proxy for mass specific metabolic rate, which can be converted into equivalent oxygen consumption rates (FMR_mass-specific_; oxygen consumption per kilogramme of body mass per hour) using the coefficients from the linear term of the statistical calibration for Atlantic cod given by (Chung *et al*., 2019*b*):


(3) \begin{equation*} {\mathrm{FMR}}_{\mathrm{mass}-\mathrm{specific}}=\frac{\left({C}_{\mathrm{resp}}-0.041\right)}{0.000971}\ \left(\mathrm{in}\ \mathrm{units}\ \mathrm{of}\ \mathrm{mg}{\mathrm{O}}_2{\mathrm{kg}}^{-1}{\mathrm{hr}}^{-1}\right) \end{equation*}

The statistical calibration for Atlantic cod is used here because there are no calibrations for wrasses. This is unlikely to cause any large implications as it has been shown that there is not a strong species-specific relationship with statical calibrations (Alewijnse, 2023; Groot  *et al*., 2017; Priester *et al*., 2024).

### Ontogenetic variation in FMR

The metabolic rate of an organism is expected to vary according to its mass, M, and temperature (in Kelvin), *T*, as: (Brown *et al*., 2004)


(4) \begin{equation*} \mathrm{Organism}\ \mathrm{Metabolic}\ \mathrm{Rate}={B}_0{M}^{\alpha }{e}^{-E/ kT} \end{equation*}

*B_0_* is a normalization constant and *α* is the allometric scaling exponent of body mass. *E* is the activation energy (or thermal sensitivity) of metabolism and *k* is Boltzmanns’s constant (8.62 × 10^−5^).

Wrasse conduct ontogenetic depth migrations with young individuals present in warm, surface waters prior to moving to deeper cooler waters with increasing age. Consequently, mass and temperature effects on field metabolism cannot be uniquely resolved. Instead, we fit equation (4) to observed FMR data, setting B_o_ as the FMR recorded at the median mass for each species. We use non-linear least squares curve fitting to explore whether coefficients α and *E* in the best fitting model differ from predictions of strict metabolic theory (α = −0.25, E = 0.65 eV (Brown *et al*., 2004)). Shallow coefficients imply dampening of thermal and body size effects on metabolism while steep coefficients imply additional behavioural or physiological energetic changes co-incident with ontogenetic and/or thermal habitat shifts.

Finally, we assess whether location (Dorset or Skye) influences expressed FMR through generalized linear models restricted to individuals with larger body sizes. Restricting modelling to largest body sizes where fish have already conducted ontogenetic depth migrations was conducted to reduce the influence of covarying mass and temperature predictor variables. Threshold body sizes for inclusion were selected based on observed body mass–temperature covariance (Fig. 5). Models were fitted using INLA (r-INLA) (Rue *et al*., 2009). All continuous variables were *z*-score transformed prior to modelling to have zero mean and unit variance. Factorial effects of species and location were included as additive and interactive terms, and model comparisons performed via DIC scores. Model diagnostics were evaluated graphically through residual analysis including Q-Q plots, Cook’s distance and residuals versus fitted values plots.

## Results

**Figure 2:**
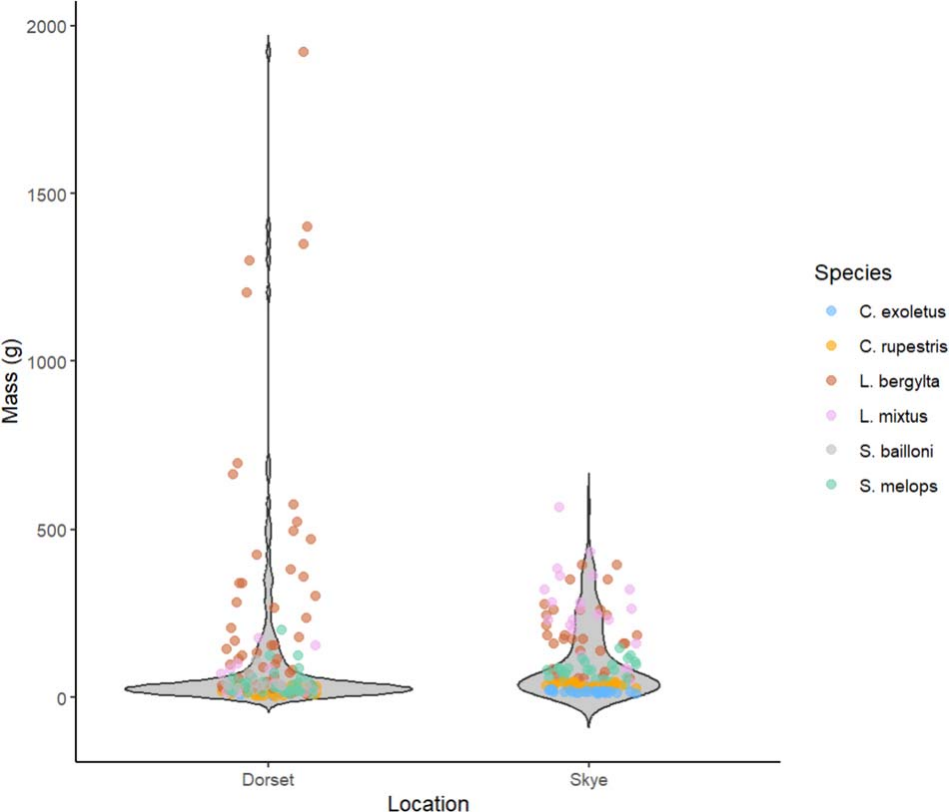
Body mass (g) of individuals plotted by location of capture. Coloured points show the raw data and are coloured by species. The grey shading in the violin plot shows the relative abundance of wrasse for a given body mass.

The range of body sizes sampled varied between locations, generally a greater range of body sizes were sampled in Dorset than in the Isle of Skye (Fig. 2). This reflected different methods of sample collection as Dorset fish were sampled before size-based sorting for individuals for fishers to retain while Skye fish were sampled after sorting. Largest individuals were constrained as: *C. exoletus*: 20 g, *C. rupestris*: 30 g, *L. bergylta*: 300 g, *L. mixtus*: 150 g, *S. melops*: 75 g, resulting in sample numbers as presented in Table 1. We successfully recovered stable isotope data from 290 individual fish from five species (excludes *S. bailloni*): 139 from Skye and 151 from Dorset. δ^18^O and δ^13^C values of otolith aragonite varied among species and location (Fig. 6, Supplementary Table S1).

**Table 1:** Estimates of mass scaling coefficient (a) and thermal sensitivity (*E*) terms recovered from fitting equation (4) to mass and otolith-inferred FMR and experienced temperature data

**Species**	***n***	**α**	SE	***E (eV)***	SE
*C. exoletus*	41	**−0.75**	0.24	−0.63	2.83
*C. rupestris*	66	**−0.84**	0.09	**−6.8**	2.4
*L. bergylta*	83	**−0.42**	0.03	**−5.5**	1.5
*L. mixtus*	42	**−0.6**	0.04	−4	1.9
*S. bailloni*	13	**−0.39**	0.07	0.62	1.2
*S. melops*	58	**−0.37**	0.04	−0.58	1.2

Values in bold indicate fitted estimates >2 SE away from predictions of strict metabolic theory (i.e. –0.25 for a and −0.65 eV for *E*). Fitted curves visualized in Fig. 5.

### Experienced temperature

Time-averaged temperatures reconstructed from otolith δ^18^O (equation (1)) ranged from 7.8 to 20.4°C. Median temperatures were 10.5°C in Skye (observed range: 7.8–14.5°C) and 14.1°C in Dorset (observed range: 9.0–20.4°C), (see Supplementary Table S1). Despite potential limitations introduced through the necessity to make assumptions about the isotopic composition of the seawater at the sampling locations (discussed in the final section). The reconstructed temperatures are consistent with the expected average temperatures of the locations, and each species reflects the predicted ~2°C difference in temperature between locations (watertemperature.info, 2024).

### Ontogenetic description of thermal habitat use in Dorset

**Figure 3:**
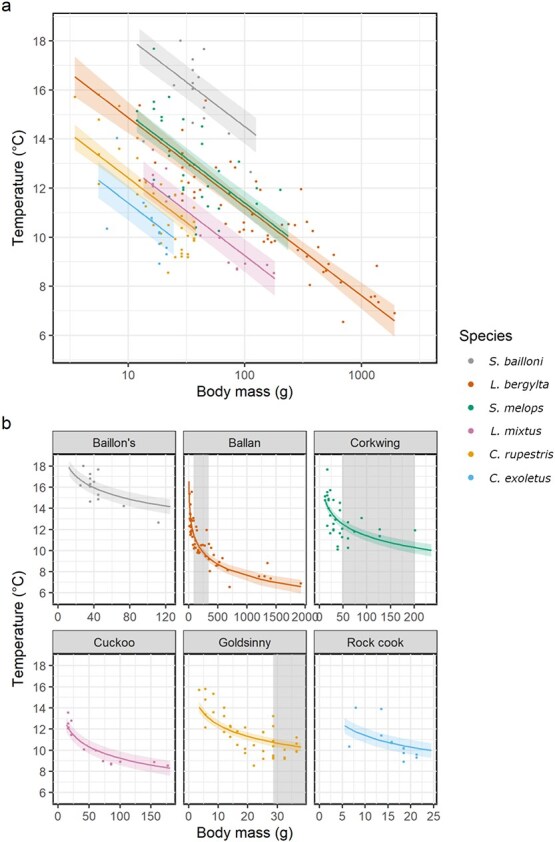
Predicted effects (with shaded 95% CI) of log_10_(body mass) (g) and wrasse species on individual mean otolith-derived experienced temperatures (°C) in Dorset only. Coloured points show the raw data. In plot a, body mass is shown on the log_10_ scale to better enable interspecific comparisons, whereas plot b is shown on the linear scale to better represent ontogenetic shifts in experienced temperature. Grey shading represents retainable sizes for each species (calculated using length–weight transformations from minimum and maximum conservation reference sizes from Southern IFCA’s district).

Individual mean otolith-derived experienced temperatures for wrasse from Dorset ranged from 9°C (*L. bergylta*) to 20.4°C (*S. bailloni*). Experienced temperature varied significantly with log_10_(body mass) and between species (Fig. 3, Supplementary Table S2). Taking these sources of variation into account, the temperatures experienced by *S. bailloni* were significantly higher than all other wrasse species for a given body mass within their size range (Tukey-adjusted pairwise comparisons, all *P* < 0.05, Supplementary Table S3). The temperatures experienced by *C. exoletus* over their range of sampled body sizes in Dorset were significantly lower than all other species (*P* < 0.05) except *C. rupestris* (Fig. 3, Supplementary Table S3). *Labrus mixtus* and *S. melops* exhibited similar experienced temperatures for their common body mass range, as did *C. rupestris* and *L. mixtus* wrasse (Fig. 3). For all species, experienced temperature declined with increasing body mass (Fig. 3). There was very limited overlap in experienced temperature between *L. bergylta* wrasse above the body mass estimated from the maximum CRS, and those below the body mass estimated from the minimum CRS (Fig. 3).

### Ontogenetic variations in FMR

Individual estimates for mass-specific oxygen consumption ranged from 14.4 (*C. rupestris*, Skye) to 390.3 mgO_2_ kg^−1^ h^−1^ (*C. rupestris*, Dorset); (equations (2) and (3)). Estimated mass-specific FMR decreased systematically through ontogeny for all species (Fig. 5). Variations in FMR were well fitted by exponential models with temperature and body size as predictors. Fitted scaling coefficients are shown in Table 1. In all species, fitted allometric scaling exponents are significantly larger (more negative) than −0.25. Fitted thermal sensitivity estimates are within error of −0.65 eV for *C. exoletus* and *S. melops*, but greater (more negative) than −0.65 eV for all other species.

### Variation in FMR within larger individuals

The best fitting INLA model describing mass and temperature effects on FMR in large individuals contained additive effects for species and common effects of mass, temperature and location:


(5) \begin{align*} \mathrm{lnO_2}\ \mathrm{consumption\ (mgO_2\ kg^{-1}\ h^{-1} )} \sim z \mathrm{(ln\ mass)}\notag \\ +\, z \mathrm{(inverse \ temperature)} + \mathrm{Species} + \mathrm{Location}. \end{align*}

The model (equation (5)) showed no difference in the relationships between mass, temperature and species between the two locations. Full-model comparison outputs are shown in the supplementary materials (Table S3) and the best fitting model is summarized in Table 2. The common thermal sensitivity was −1.64 eV (95% CI: −2.1 to −1.2 eV). The common allometric mass slope (α) was −0.53 (95% CI: −0.76 to −0.3). Species intercepts (*Bo*) for mass-specific oxygen consumption rates (i.e. relative metabolic level) of *L. bergylta*, *L. mixtus* and *S. melops* were higher than those of *C. exoletus* and *C. rupestris*.

**Table 2:** Fixed effect terms for the best fitting INLA model of variation in FMR in large individuals

	**Mean**	**0.025CI**	**0.975 CI**	**Rescaled**
Intercept (*C. exoletus*)	3.62	3.21	4.03	37.4 mgO_2_ kg^–1^ hr^–1^
Mass effect (z_lnMass)	−0.65	−0.94	−0.37	−0.53
Temperature effect (*z* inverse temperature)	−0.40	−0.52	−0.28	−1.66 eV
*C. rupestris*	0.08	−0.19	0.35	40.5 mgO_2_ kg^–1^ hr^–1^
*L. bergylta*	1.68	0.89	2.47	200.5 mgO_2_ kg^–1^ hr^–1^
*L mixtus*	0.93	0.31	1.55	94.8 mgO_2_ kg^–1^ hr^–1^
*S. melops*	0.90	0.36	1.44	92.2 mgO_2_ kg^–1^ hr^–1^
Location: Skye	0.05	−0.16	0.26	

Model-predicted oxygen consumption rates were estimated for a common temperature (10.5°C, the median temperature across all observations) and for both species’ median mass and common mass (81 g). Across all species, FMR estimates from Dorset and Skye were similar when corrected to a common temperature and body size (Fig. 6).

## Discussion

This study characterized inter- and intra-specific variation in experienced temperature and FMR for commercially important temperate wrasse species. We found evidence of consistent ontogenetic shifts in experienced temperature within location. In all species except *C. exoletus,* FMR declined with body size and experienced temperature at a greater rate than expected from considerations of thermodynamics and allometric body size scaling. This implies ontogenetic habitat shifts are associated with behavioural reductions in metabolic rate, which represent either activity levels and/or specific dynamic action. The interactive nature of body size, temperature and behaviour complicate efforts to deconvolve mass, temperature and potential location effects on species’ FMRs.

### Thermal habitat use of Dorset wrasse

There was clear interspecific separation in the temperatures experienced by wrasse across their common ranges of sampled body sizes. *S. bailloni* wrasse were found at the warmest temperatures for a given body size, which is unsurprising given that the English Channel is believed to be close to the northern limit of this species’ range (Dunn and Brown, 2003), so they may preferentially inhabit the warmest available waters. This is in clear contrast to the other species studied here, which are all commonly found across a broad range of more northerly and southerly latitudes (Henderson, 2014; Skiftesvik *et al*., 2014*b*; Froese and Pauly, 2018). The geographic range of *L. bergylta* wrasse, uniquely for this group, extends further south into Morocco (Porteiro *et al*., 1996; Pita and Freire, 2011; Villegas-Ríos *et al*., 2013), so is likely to be one of the more warm-adapted of the remaining temperate wrasse species. This corresponds with the findings reported here, which show that, across their overlapping size ranges, *L. bergylta* experienced higher temperatures than all remaining species apart from *S. melops*. *S. melops* experienced similar temperatures to *L. bergylta* wrasse of the same body size, potentially because they exhibit a preference for shallower waters, often <5 m depth (Darwall *et al*., 1992; Halvorsen *et al*., 2020; Henly *et al*., 2021), so are likely to experience warmer temperatures than other species that do not show the same affinity to shallow, sheltered water. Of the remaining wrasse species, *C. exoletus* experienced the lowest temperatures across their shared body sizes with other species and *L. mixtus* experienced the highest, although neither were statistically different from the temperatures experienced by *C. rupestris*. Relatively little is known about the habitat preferences of these species; however, previous work has shown they have varying relative abundances across fairly small spatial scales (Skiftesvik  *et al*., 2014*b*; Halvorsen *et al*., 2020; Henly *et al*., 2021). Both *C. rupestris* and *C. exoletus* are reported to prefer more exposed locations with more water movement compared to *S. melops* (Skiftesvik  *et al*., 2014*b*; Henly *et al*., 2021), and *L. mixtus* occupy a much broader range of depths, down to 200 m (Henderson, 2014; Froese and Pauly, 2018), which could explain the relative differences in temperatures experienced by these species in comparison to those already discussed.

### Intraspecific ontogenetic variation in thermal habitat

There is currently limited evidence of how the thermal preferences of the different wrasse species change throughout ontogeny, so these data provide a key insight. The systematic reduction in otolith-inferred temperature during early ontogeny observed for all species in Dorset (where the sampled body size range was sufficiently large) implies that wrasse migrate from warm and shallow nursery habitats to their adult habitat. For example, *L. bergylta* fish larger than 18–20 cm no longer experience the range of temperatures experienced by the smaller fish (Fig. 3). Limited previous observations support our inferences (Dipper *et al*., 1977; Darwall *et al*., 1992; Sayer *et al*., 1993). For example, Dipper *et al*., (1977) found juvenile *L. bergylta* (~1 year, <10 cm) in intertidal pools, whereas individuals >20 cm were only seen in the sub-littoral. Individuals between 10 and 20 cm were found in both habitats (Dipper *et al*., 1977). Similarly, juvenile *C. rupestris* are most commonly observed in shallower (2–7 m) depths, and are not observed at the deeper sites more commonly inhabited by adults (Quinard and Pras, 1984; Sayer *et al*., 1993). However, for many of the wrasse species studied here more samples would be needed to ensure the observed ontogenetic patterns are not a result of uneven sampling effort across the range of body sizes. Nevertheless, to our knowledge, these observations are some of the first quantitative evidence that ontogenetic shifts in thermal habitat are common across co-existing wrasse species in coastal waters.

### FMR

**Figure 4:**
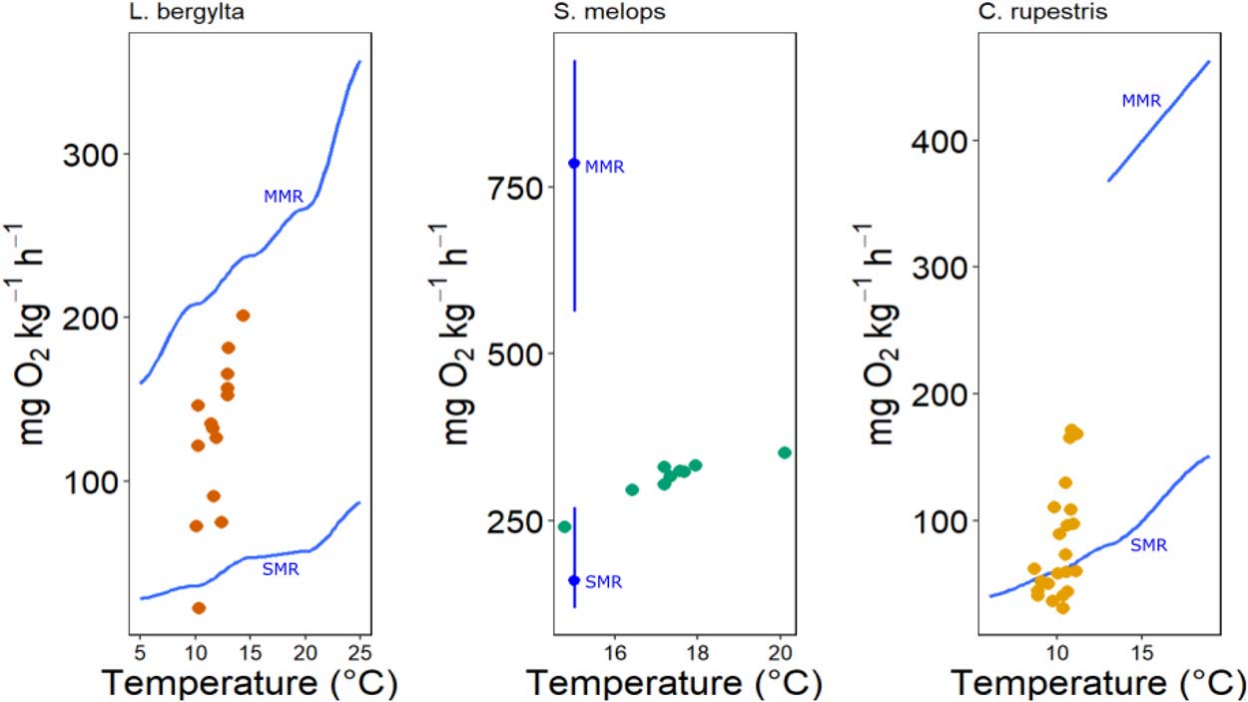
Comparison between laboratory-determined SMR and MMR and field inferred FMR (coloured points) for three wrasse species. *Labrus bergylta* SMR and MMR data from Yuen *et al*., (2019), *S. melops* data from Norin *et al*., (2021) and *C. rupestris* data from Sayer and Davenport (1996) and Perry *et al.,* (2024).

The FMR estimates obtained in this study represent the first estimates of metabolic rate for several of these commercially important temperate wrasse species. We are aware of metabolic data in the form of laboratory-determined oxygen consumption rates for three of the wrasse species considered here. The estimated mass-specific oxygen consumption values for *L. bergylta*, *S. melops* and *C. rupestris* are comparative to respirometry data from Yuen *et al*. (2019), Sayer and Davenport (1996), Perry *et al*. (2024) and Norin *et al*. (2021), respectively (Fig. 4). In each case, FMR estimates at equivalent body size and temperature from otoliths lie between laboratory-determined standard metabolic rate (SMR) and maximum metabolic rate (MMR). We are therefore encouraged that assumptions inherent in estimating C_resp_ values and conversion to equivalent oxygen consumption rates are supported where laboratory-determined comparative data are available.

### Interspecific variation in FMR

For a common temperature and body size, *L. bergylta*, *L. mixtus, S. bailloni* and *S. melops* had higher mass-specific FMR than *C. rupestris* and *C. exoletus*. Observed differences in FMR are relatively small compared to uncertainties in model fitting, however, reflecting both unbalanced sample numbers and non-independence of mass and temperature.

Median metabolic rates at body sizes above the migration body size threshold ranged from 44 mgO_2_ kg^−1^ hr^−1^ at 7.5 ±1.3°C in *L. mixtus* to 188 mgO_2_ kg^−1^ hr^−1^ at 12 ±0.5°C in *S. melops*. Despite having relatively high FMR when scaled to a common body size, realized FMR in larger sampled individuals of *L. mixtus, L. bergylta* and *C. rupestris* from around Skye are <100 mgO_2_ kg^−1^ hr^−1^. It has been assumed that wrasse species with small home range sizes (Sjolander *et al*., 1972; Potts, 1984, 1985; Sayer, 1999; Villegas-Ríos *et al*., 2013; Skiftesvik  *et al*., 2014*b*; Halvorsen *et al*., 2021) are fairly sedentary, yet there is limited quantitative evidence of relative inter-specific activity levels or food requirements. The FMRs presented here fall within laboratory-inferred measures of SMR and MMR, and support inferences of relatively low activity levels and corresponding FMRs for all sampled wrasse species, but particularly for larger adult individuals of *L. mixtus* and *C. rupestris.*

### Ontogenetic variation in FMR

**Figure 5:**
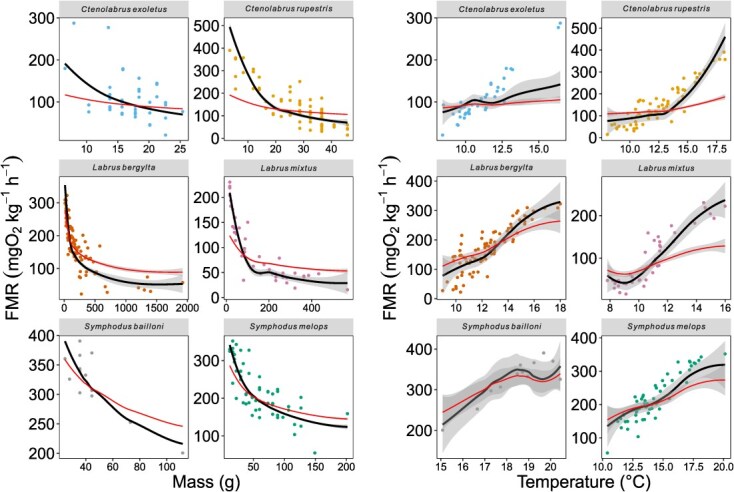
Ontogenetic variations in otolith-inferred FMR relative to mass (left panels) and otolith-derived temperature (right panels). Black lines show best fitting exponential models (FMR ~ B_o_ *Mass^α^ * e^–E/kT^), where B_o_ is the FMR recorded at the median mass for each species. Red lines show variation in FMR predicted from strict metabolic theory (α = −0.25, *E* = −0.65 eV).

Exponential models with mass and temperature as predictors successfully capture observed among-individual variation in FMR across body sizes. However, ontogenetic trends in FMR demonstrate greater reductions in FMR than are predicted from metabolic theory (Brown *et al*., 2004) based on the observed body mass and temperature (Fig. 5). We infer that the ontogenetic thermal habitat changes from warm to cooler habitats identified in this study in all sampled wrasse species are associated with an additional decrease in activity. Ontogenetic habitat shifts to deeper water (Dipper *et al*., 1977; Darwall *et al*., 1992; Sayer *et al*., 1993) may be associated with lower energetic costs through differences in foraging strategy and movement behaviour and/or moving below surface waters with high wave energy conditions. Dramatic reductions in energy use are reported for coral reef fish undergoing transition from pelagic to reef-associated lifestyles (Downie *et al*., 2024), and we infer that analogous energetic consequences occur during the transition from pelagic foraging to reef-associated territorial place-holding behaviours in temperate wrasse. Energetic effects of habitat shifts are more strongly seen in *L. mixtus*, *C. rupestris* and *C. exoletus*, and less emphasized in *L. bergylta, S. bailloni* and *S. melops*. In any case otolith isotope analyses identify hitherto unrecognized, or unquantified, energetic variations, and presumably associated reductions in feeding rates through ontogeny, which may have implications for size-based performance of wrasse as clearnerfish in aquaculture settings.

**Figure 6:**
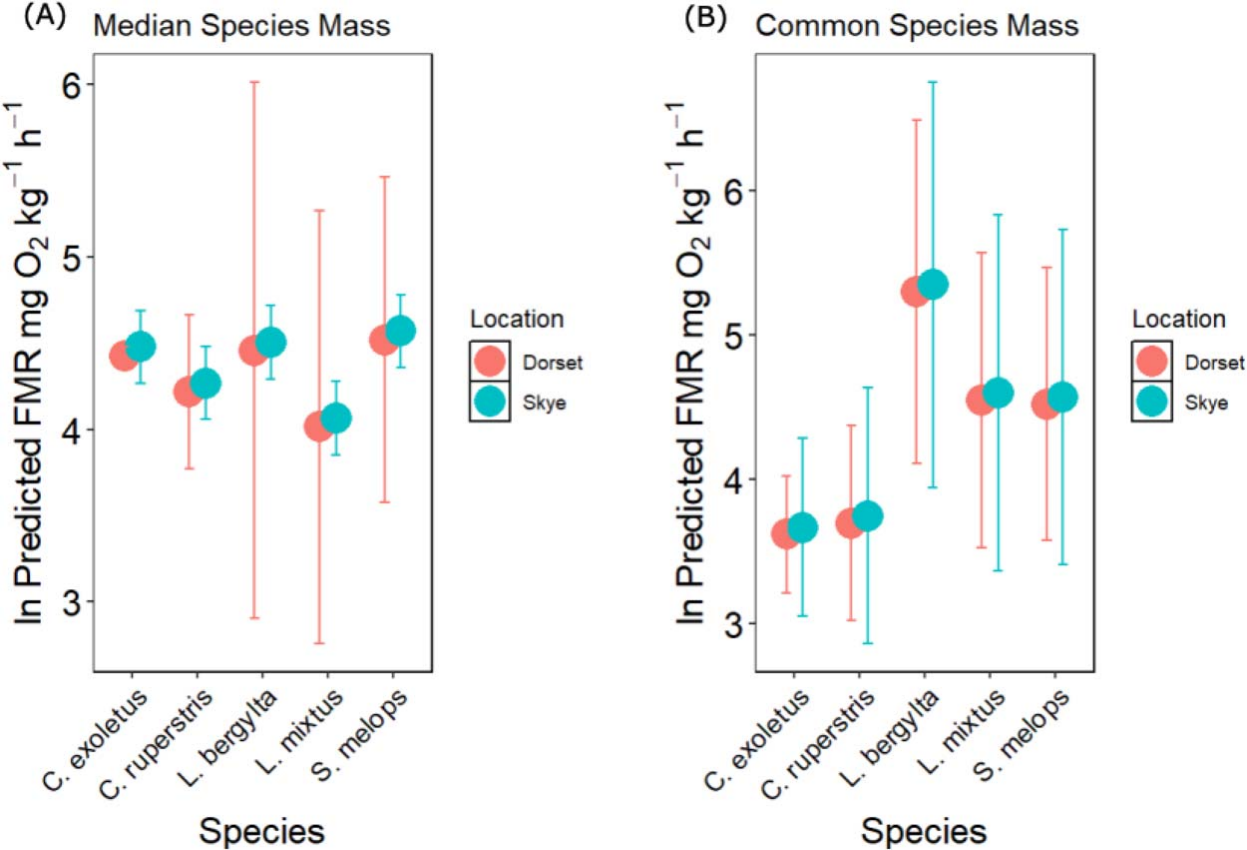
INLA-predicted mass-specific FMR (equation (5)) for large body-sized wrasse from Dorset and Skye predicted for a common temperature of 10.5°C, and (A) the median sampled mass for large individuals of each species and (B) a single common mass of 81 g.

### Implications for wrasse fisheries and use in salmon aquaculture

The ontogenetic thermal habitat preferences of wrasses demonstrated in this study could have potential implications for the effectiveness of conservation measures designed to limit the impacts of live wrasse fisheries. In many locations where a live wrasse fishery exists, minimum and maximum CRS have been set to help ensure the live wrasse fishery does not remove too large a proportion of the sexually mature individuals within the population (SIFCA, 2021*b*, 2021a; Smith and Henly, 2021). For *L. bergylta*, which is a protogynous (female to male) hermaphrodite, the minimum and maximum CRS in Dorset have been set at 18 and 28 cm, respectively, to help protect smaller mature females (<18 cm) and larger mature males (>28 cm). However, our observations suggest that *L. bergylta* >28 cm occupy a different thermal habitat and energetic behaviour to smaller individuals <18 cm in length (see Figs 2 and 4). Therefore, eggs and milt of non-targeted spawning wrasse are less likely to co-occur at high concentrations over small spatial scales. If the landable wrasse (between 18 and 28 cm) are overexploited, this may leave the remaining population vulnerable, as their chances of breeding could be significantly reduced due to a mismatch in thermal habitat preferences between recently matured females <18 cm and larger mature males >28 cm.

The observed dramatic change in FMR (and associated feeding rates) with body size also has potential implications for the use of wrasse as cleanerfish in salmonid aquaculture. Higher feeding rates imply greater effectiveness as cleanerfish, potentially driving selection of the smallest landable fish large enough to avoid escapement from salmon nets (Sistiaga *et al*., 2021). As wrasse grow within salmon cages, feeding rates are speculated to drop, resulting in requirements to replace wrasse populations regularly to retain cleaning efficiency. *Labrus bergylta* show the highest energy use at a common body size for large individuals implying optimal performance as cleaner fish. However*, C. rupestris* and *L. mixtus,* and potentially *C. exoletus*, displayed relatively dramatic decreases in energy use with increasing body size, and *C. rupestris* individuals between 10 and 20 g show the greatest elevation of energy use above metabolic predictions. Providing small size does not allow escape from salmon cages, *C. rupestris* individuals may perform optimally in salmon cages at body masses <20 g.

### Otolith method: opportunities and limitations

The C_resp_ method fills an important methodological gap in the study of FMR across vertebrates, with the ability to estimate FMR in aquatic organisms (Treberg *et al*., 2016; Yuen *et al*., 2019; Alewijnse, 2023). Natural sampling creates a number of limitations. The samples used in this study were collected in mid-September. Otolith sampling integrated several months of data, essentially constraining energy use over spring–summer feeding periods. Multiple samples throughout the year would allow us to study seasonal variation. To reconstruct experienced temperature, we make assumptions about the isotopic composition of the seawater (Morissette *et al*., 2023) based on available measured δ^18^O values within 1.5 degrees of latitude and longitude, and at salinities >30 a.s.u., but without specific measurements, estimates are uncertain. Similarly, as we did not take *in situ* measurements of seawater temperature across a range of depths in each location (potentially missing temperature stratification). We rely here on sea surface temperatures to validate the experienced temperature results. CTD (conductivity, temperature, depth) sea temperature data from the Western Channel Observatory’s coastal L4 sampling station, located 10 nautical miles southwest of Plymouth, indicated differences in sea temperature of <2°C across the 0- to 50-m depth range (WCO, 2020). Assuming our sampling sites experience similar temperature stratification profiles, we can conclude the inferred temperatures lie within the temperatures expected to be experienced by wrasse in Dorset and Skye.

Estimates of FMR based on otolith δ^13^C values are subject to similar assumptions around the isotopic compositions of carbon sources (diet and DIC). We have measured δ^13^C_diet_ for both populations, but there are only mean values for each species for the Skye wrasse. As the carbon contributing to otolith growth is mostly derived from external seawater, C_resp_ values are relatively insensitive to small changes in δ^13^C_diet_ values. The δ^13^C of seawater was not measured at the time of sampling and was estimated as 1‰ based on observations from the North Sea (Burt *et al*., 2016). Inferred C_resp_ values are much more sensitive to variations in δ^13^C values of DIC. Accordingly, it is likely that we did not capture true variations in δ^13^C_DIC_. The relationship between C_resp_ values and oxygen consumption rates is assumed to be fixed among species providing the concentration ratio of O_2_ and CO_2_ in ambient water is essentially constant and most CO_2_ is lost at the gills. Additional experimental and theoretical work is needed to constrain calibrations and to evaluate the extent to which a common calibration can be applied across taxa. Nonetheless, we point to the close correspondence between laboratory-derived respirometry and our otolith-inferred FMR estimates as supporting evidence for the efficacy of common calibration equations (Chung *et al*., 2019*b*; Groot  *et al*., 2024).

## Conclusions

This study demonstrates the insight that can be gained from stable isotope analysis of otolith aragonite. All sampled wrasse species display ontogenetic trends in thermal preference, with clear reduction in preferred temperatures at threshold body sizes. Mass specific energy use also varies through ontogeny and across species. Energy use trends are relatively well described by canonical metabolic scaling exponents for four species, but *C. rupestris* and *L. mixtus* display energetic changes above those predicted by changes in temperature and body size, suggesting additional reductions in activity associated with habitat shifts to deeper water in these two species.

Our results could have important implications for fisheries management and conservation in terms of selective capture and breeding programmes. We identify potential conflicts between landing size limits and size-based thermal habitat separation in sexually dimorphic wrasse, and identify species and life stages with higher mass-specific energy use (and therefore feeding rates). While *C. rupestris* showed the highest FMR, this was only for sizes <20 g; therefore, overall, we would suggest *L. bergylta* is the most commercially viable species, as it shows the highest energy use at a common body size for large individuals implying optimal performance as cleaner fish.

More broadly, this study shows that field-based, context-specific ecophysiological data and thermal preference data can be obtained from retrospective otolith isotope analyses at relatively low cost, offering potential for physiology-informed fisheries conservation and management.

## Supplementary Material

Web_Material_coag026

## Data Availability

The data underlying this article are available in the article and in its online supplementary material: https://github.com/EBall98/Stewart-Ball-Trueman-and-Stevens-2026---wrasse.git
