# Supplementary material for: Ontogenetic thermal and metabolic patterns guide physiologically informed, size-based fishery management
Source: Conserv Physiol. 2026 Apr 22;14(1):coag026. doi: 10.1093/conphys/coag026 (PMC13102501; doi:10.1093/conphys/coag026)
Supplement: Web_Material_coag026 [file web_material_coag026.zip › Supplementary_Material_20260107 (1).pdf]

Supplementary Material

Supplementary Table S1: Summary information for six wrasse species including number of individuals sampled, median otolith $\delta^{18}O$ and $\delta^{13}C$ values (in per mille, ‰), median calculated experienced temperature (°C) and mass-specific oxygen consumption (mg02kg-1hr-1) for each species and location.

Species	**Location**	**n**	**$\delta^{13}C_{oto}$ median (IQR)**	**$\delta^{18}O_{oto}$ median (IQR)**	**Temperature median (min, max)**	**Oxygen consumption $mg0_2kg^{-1}hr^{-1}$ median (min, max)**
S. bailloni	Dorset	13	**-7.4** (0.7)	**0.08** (0.4)	**19** (15.1, 20.4)	**330** (200, 390)
L. bergylta	Dorset	54	**-5.2** (2.4)	**1.26** (1.6)	**13** (9, 18)	**192** (27, 323)
	Skye	29	**-3.9** (0.6)	**1.5** (0.3)	**10.8** (9.8, 13.8)	**143** (20, 230)
S. melops	Dorset	32	**-6.5** (1.9)	**1.1** (0.3)	**15** (12.5, 20)	**250** (160, 350)
	Skye	26	**-4.3** (1.9)	**1.8** (0.4)	**12.9** (10.4, 14.7)	**169** (55, 228)
L. mixtus	Dorset	14	**-4.4** (1.9)	**1.4** (0.7)	**12.4** (11, 16)	**155** (66, 231)
	Skye	28	**-2.4** (1.1)	**1.8** (0.4)	**9.5** (7.8, 12.3)	**48** (16, 176)
C. rupestris	Dorset	39	**-4.7** (2.2)	**1.1** (0.5)	**13.6** (11, 18)	**166** (87, 390)
	Skye	27	**-2.6** (1)	**1.6** (0.26)	**10.5** (8.1, 13.6)	**62** (14, 171)
C. exoletus	Dorset	12	**-4.34** (1)	**1.3** (0.25)	**12.6** (11.4, 16.5)	**151** (111, 288)
	Skye	29	**-3** (0.3)	**1.6** (0.2)	**10.2** (8.4, 12.6)	**82** (21, 135)

Supplementary Table S2: Parameter estimates (and standard errors) for drivers of variation (Log_{10}(Body Mass) = Mass; and Species = Sp_{xx}) in otolith-derived experienced temperatures in candidate and null models. M_{BEST} represents the candidate model with the lowest AIC, M_{FINAL} represents the selected, most parsimonious model and M_{NULL} is the null model. k is the degrees of freedom in each model, LL is the log-likelihood, AIC is the absolute Akaike's Information Criterion, and ΔAIC is the relative difference in AIC compared to $M_{BEST.}$ The Species factor (Sp_{XX}) has the following levels LB: *L. bergylta*; SM: *S. melops*; LM: *L. mixtus*, CR: *C. rupestris*; CE: *C. exoletus*. *S. bailloni* (SB) was used as the base level of the species factor.

	β_0	Mass	Sp_{LB}	Sp_{SM}	Sp_{LM}	Sp_{CR}	Sp_{CE}	Mass: Sp_{LB}	Mass: Sp_{SM}	Mass: Sp_{LM}	Mass: Sp_{CR}	Mass: Sp_{CE}	k	LL	AIC	ΔAIC
M_{BEST}	26.69 (3.25)	-6.67 (1.99)	-9.25 (3.30)	-8.09 (3.42)	-8.90 (3.54)	-8.23 (3.37)	-9.06 (4.16)	3.54 (2.01)	3.07 (2.11)	2.26 (2.16)	1.12 (2.11)	0.82 (2.97)	13	-254.04	534.08	0
M_{FINAL}	21.76 (0.52)	-3.63 (0.24)	-3.26 (0.40)	-3.11 (0.40)	-5.26 (0.47)	-5.72 (0.40)	-6.74 (0.50)	–	–	–	–	–	8	-261.78	539.56	5.48
M_1	15.89 (0.53)	–	-5.10 (0.59)	-2.84 (0.63)	-5.36 (0.73)	-4.41 (0.61)	-5.13 (0.76)	–	–	–	–	–	7	-335.48	684.95	150.87
M_2	15.80 (0.51)	-2.43 (0.29)	–	–	–	–	–	–	–	–	–	–	3	-346.53	699.05	164.97
M_{NULL}	11.77 (0.19)	–	–	–	–	–	–	–	–	–	–	–	2	-375.54	755.07	220.99

Supplementary Table S3: Tukey post-hoc estimates, standard errors, z values and p values (adjusted for multiple comparisons) for all combinations of species comparing otolith-derived experienced temperature. SB: *S. bailloni*, LB: *L. bergylta*; SM: *S. melops*; LM: *L. mixtus*, CR: *C. rupestris*; CE: *C. exoletus*. *, **, *** denote significant differences ($p < 0.05$, $p < 0.01$, $p < 0.001$) in otolith-derived experienced temperatures between species.

	Estimate	SE	z	P_{adj}	
SB:LB	-3.26	0.40	-8.23	<0.001	***
SM:SB	-3.11	0.40	-7.75	<0.001	***
LM:SB	-5.26	0.47	-11.19	<0.001	***
CR:SB	-5.72	0.40	-14.3	<0.001	***
CE:SB	-6.74	0.50	-13.48	<0.001	***
SM:LB	0.15	0.31	0.48	0.99	
LM:LB	-2.00	0.38	-5.21	<0.001	***
CR:LB	-2.46	0.33	-7.45	<0.001	***
CE:LB	-3.48	0.45	-7.71	<0.001	***
LM:SM	-2.15	0.39	-5.48	<0.001	***
CR:SM	-2.61	0.30	-8.73	<0.001	***
CE:SM	-3.63	0.42	-8.59	<0.001	***
CR:LM	-0.46	0.39	-1.19	0.84	
CE:LM	-1.48	0.49	-3.01	0.03	*
CE:CR	-1.02	0.40	-2.53	0.11	
